# Differential Modulation of GLP-1R by Dietary Ginsenosides Points to a Putative Extracellular Allosteric Site

**DOI:** 10.3390/ijms27125630

**Published:** 2026-06-22

**Authors:** Ayelet Caspi, Netaly Khazanov, Aharon Helman, Hodaya Lankry, Berta Levavi-Sivan, Hanoch Senderowitz, Zohar Kerem

**Affiliations:** 1 Institute of Biochemistry, Food Science and Nutrition, Robert H. Smith Faculty of Agriculture, Food and Environment, The Hebrew University of Jerusalem, Rehovot 7610001, Israel; ayelet.bait-halachmy@mail.huji.ac.il (A.C.); aharon.helman@mail.huji.ac.il (A.H.); 2 Department of Chemistry, Bar-Ilan University, Ramat Gan 5290002, Israel; netalyk@gmail.com (N.K.); hsenderowitz@gmail.com (H.S.); 3 Department of Animal Sciences, Robert H. Smith Faculty of Agriculture, Food and Environment, The Hebrew University of Jerusalem, Rehovot 7610001, Israelberta.sivan@mail.huji.ac.il (B.L.-S.)

**Keywords:** GLP-1R, ginsenosides, Panax ginseng, allosteric modulation, computational analyses

## Abstract

The glucagon-like peptide-1 receptor (GLP-1R) is a class B G protein-coupled receptor (GPCR) central to metabolic regulation, and its potential modulation by dietary phytochemicals is increasingly recognized as physiologically relevant. Understanding how such compounds interact with GLP-1R is important for clarifying mechanisms that may contribute to gut-to-brain signaling. In this study, we examined three structurally related dietary ginsenosides, Rg1, Rg2, and Rg3, as potential modulators of GLP-1R using luciferase reporter assays and computational analyses. Despite sharing similar molecular weights, a common dammarane scaffold, and comparable sugar moieties, the three ginsenosides displayed distinct effects on GLP-1R activity: Rg2 and Rg3 potently reduced receptor activation in a dose-dependent manner when co-administered with Exendin-4, whereas Rg1 had minimal effect. Computational screening of the GLP-1R structure for binding sites identified a putative extracellular pocket on the protein that can accommodate these compounds, while molecular docking and binding free energy calculations provided predicted affinities qualitatively reflecting the phytochemicals’ experimental activities. These findings point to a plausible extracellular mechanism through which dietary ginsenosides may influence GLP-1R responsiveness at the intestinal interface. Our results point to the possibility that non-absorbed phytochemicals can differentially modulate gut-expressed receptors, suggesting a novel pathway for dietary signaling relevant to ethnopharmacology and metabolic health.

## 1. Introduction

G protein-coupled receptors (GPCRs) constitute the largest and most versatile family of membrane proteins and mediate a vast array of physiological responses through ligand-activated signal transduction [1]. While traditional pharmacological strategies have focused on orthosteric sites, recent research has highlighted the importance of allosteric modulation, in which ligands bind to topographically distinct regions to influence receptor activity, offering opportunities for increased selectivity and reduced off-target effects [2]. These allosteric sites, often located in extracellular loops or transmembrane regions, act as dynamic regulatory elements that govern receptor conformation and signaling [3].

One prominent example of a GPCR that has attracted significant therapeutic interest is the glucagon-like peptide-1 receptor (GLP-1R), a Class B GPCR. GLP-1R plays a critical role in the regulation of glucose homeostasis and appetite, making it a valuable target in the treatment of metabolic disorders such as type 2 diabetes and obesity. Its endogenous ligand, glucagon-like peptide-1 (GLP-1), is a 30-amino acid peptide that adopts an α-helical conformation upon receptor binding, engaging both the extracellular domain and the transmembrane core of GLP-1R [4]. In recent years, several studies have uncovered non-peptidic small-molecule positive allosteric modulators (PAMs) that potentiate GLP-1R signaling by stabilizing active receptor conformations or enhancing peptide binding [2,5,6,7]. Additionally, studies on other Class B1 GPCRs, such as the calcitonin gene-related peptide receptor, have shown that small molecules bind the extracellular domain to inhibit receptor activation and stabilize inactive conformations, illustrating the mechanistic diversity of allosteric regulation within Class B1 GPCRs [8].

Recent advances in gut physiology demonstrate the complexity of metabolic regulation in the gastrointestinal tract. Many non-nutrient phytochemicals transit the GI tract unabsorbed, allowing them to interact with the colonic environment or undergo microbial metabolism. Enteroendocrine cells (EECs), densely distributed along the intestinal lining, sense these compounds and release gut hormones such as GLP-1, which modulate systemic metabolic responses [9].

Recognizing the gut as a sensory organ underscores the importance of rapid neural pathways, in addition to slower hormonal mechanisms, in transmitting intestinal signals to the brain to regulate appetite and metabolism [10]. GLP-1R expressed on rapid vagal afferent neurons in regions such as the intestine, plays a key role in mediating satiety signals [9,11,12]. Given this intricate interplay between gut-derived signals, brain circuits, and receptor modulation, there is a growing interest in identifying natural compounds capable of influencing these pathways [9]. One such group of compounds is ginsenosides, derived from ginseng [13,14,15].

Panax ginseng is well known as a widely used traditional herbal and adaptogen and continues to gain global recognition for its medicinal properties [16]. Ginsenosides, its principal bioactive compounds, have attracted considerable attention for their antidiabetic properties. Recent studies have demonstrated that specific ginsenosides, such as Rg3, Rb1, and compound K, can significantly stimulate GLP-1 secretion from enteroendocrine L-cells, primarily via interactions with the sweet taste receptor pathway [15]. This GLP-1 secretagogue activity has been associated with improved glucose homeostasis and insulin secretion in both cellular and animal models, highlighting a link between ginsenoside structure and metabolic regulation [14]. GLP-1R in colonic epithelial cells contributes to the regulation of local cellular metabolism and maintenance of glucose homeostasis. Recent evidence indicates that impairment of GLP-1R signaling in the colon may disrupt the tissue’s metabolic adaptability to glucose load, thereby facilitating the progression of insulin resistance and type 2 diabetes mellitus [9,17]. Further, newly published analyses underscore the colonic GLP-1/GLP-1R axis as a promising pharmacological target, with potential for both therapeutic intervention and prevention strategies in diabetes management [18,19]. Given the central role of GLP-1R in mediating the incretin effect and its therapeutic relevance in diabetes, investigating whether ginsenosides also directly modulate GLP-1R function beyond stimulating hormone secretion offers a compelling avenue for uncovering novel mechanisms and potential therapeutic strategies [13].

**Figure 1:**
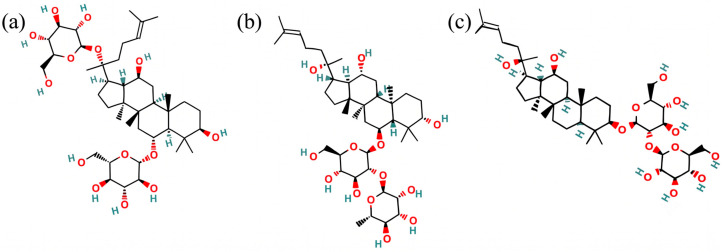
2D chemical structures of the selected ginsenosides: (**a**) Rg1 (**b**) Rg2 and (**c**) Rg3. Structures were retrieved from PubChem (https://pubchem.ncbi.nlm.nih.gov/ (accessed on 14 May 2025)).

Ginsenosides are small rigid dammarane type tetracyclic triterpenoid saponins bearing sugar moieties [20], distinguishing them structurally and functionally from the relatively large, flexible, and hydrophilic peptide ligands of GLP-1R [21,22]. They have previously been reported to stimulate intestinal GLP-1 secretion [14,15]. If these compounds also directly modulate binding of GLP to GLP-1R, when the receptor is expressed in the gut epithelium, investigating it would provide new insights not only into how ginsenosides act but also into how small dietary phytochemicals in general might engage this receptor through mechanisms distinct from those used by endogenous peptides and peptide-based therapeutics. In this study, we focus on the structurally related dietary ginsenosides Rg1, Rg2, and Rg3 (Figure 1) and explore their effects on GLP-1R. Despite their differences in sugar attachment positions, Rg1, Rg2, and Rg3 share similar molecular weights, a common dammarane backbone, and broadly comparable glycosylation patterns, making them well-suited for comparative analysis.

**Aims of this study:** The primary objective of this study was to evaluate the potential of specific phytochemicals to modulate the function of the human glucagon-like peptide-1 receptor (GLP-1R) through an integrated computational and experimental pipeline. Specifically, this work aimed to: (1) computationally screen and map potential novel allosteric binding sites within the extracellular domain of the GLP-1R; (2) predict the binding affinity of candidate ginsenosides (Rg1, Rg2, and Rg3) using molecular docking and MM-GBSA simulations; and (3) functionally validate these computational predictions in vitro using an expanded, multi-control cellular reporter system to determine the effect of the ginsenosides on receptor-mediated downstream signaling.

## 2. Results

### 2.1. Functional Assessment of Rg1, Rg2 and Rg3 as GLP-1R Modulators Using a Luciferase Reporter Assay

**Figure 2:**
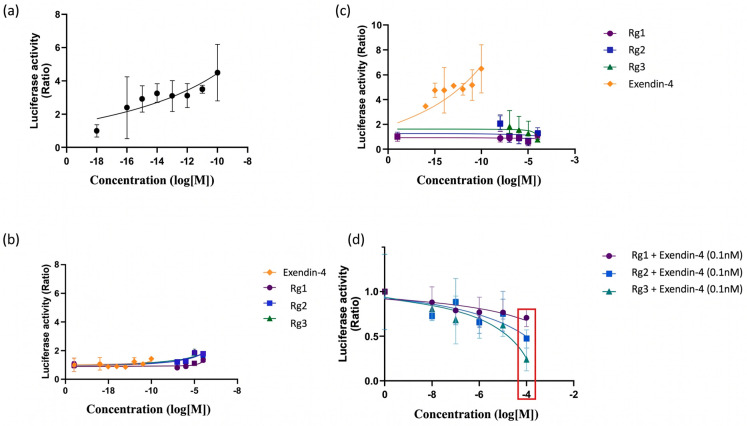
**In vitro characterization and functional validation of human GLP-1R modulation by ginsenosides.** (**a**) GLP-1R activation profile in the presence of varying concentrations of the canonical agonist Exendin-4, confirming dose-dependent cAMP-responsive element (CRE) reporter activity and system validity. (**b**) Specificity control assessing whether the tested compounds induce non-specific cellular signaling in untransfected HEK293 cells under the same assay conditions, independent of GLP-1R/pCRE-LUC transfection. (**c**) Intrinsic activation assay evaluating human GLP-1R-expressing cells treated with varying concentrations of the tested compounds alone, demonstrating an absence of direct orthosteric or partial agonism. (**d**) Functional antagonism profiling showing GLP-1R signaling activity in the presence of varying concentrations of RG compounds (Rg1, Rg2, and Rg3) co-administered with a fixed, sub-maximal concentration of Exendin-4 (0.1 nM). Data points represent mean values ± SEM.

To evaluate the modulatory potential of Rg3 and its structurally related analogs, we used an in vitro luciferase-based reporter assay in HEK293 cells transiently transfected with the human GLP-1R and a cAMP-responsive element (CRE) luciferase reporter plasmid. HEK293 cells were selected to establish a robust experimental baseline. Preliminary optimization across an alternative mammalian expression host, COS7 cells, confirmed consistent signaling trends, with HEK293 cells providing superior baseline stability and reproducibility for definitive profiling. Representative COS7 optimization data are provided in Supplementary Figure S1, which result from a significantly reduced CRE-luciferse signal in comparison with HEK293 cells (Figure 2).

To map the functional pharmacology of the ginsenosides and rule out non-specific assay artifacts, four distinct assays were performed (Figure 2). Across all assay conditions, including transfected and untransfected cells, routine light-microscopy inspection revealed no overt morphological changes or apparent loss of cell viability at the tested compound concentrations.

Figure 2a demonstrates that application of the reference peptide agonist Exendin-4 alone yielded a robust, concentration-dependent increase in luciferase expression, validating the transfection efficiency and signaling competency of the Gs-coupled cAMP reporter cascade.

To determine whether the tested phytochemicals induce non-specific cellular responses through GLP-1R-independent pathways, untransfected, null-background HEK293 cells were exposed to varying concentrations of Rg1, Rg2, or Rg3 under the same assay conditions (Figure 2b). None of the compounds induced detectable baseline deviation or signal suppression in this null-background system, suggesting that the observed modulation in GLP-1R-expressing cells was not attributable to non-specific cellular activation.

Next, to assess whether the ginsenosides possess intrinsic receptor-activating capabilities, GLP-1R-transfected cells were treated with the compounds alone in the absence of Exendin-4 (Figure 2c). No elevation in luciferase activity above baseline was observed, indicating that Rg1, Rg2, and Rg3 do not act as direct orthosteric agonists or partial agonists under these assay conditions.

Finally, to evaluate functional modulation, a fixed, sub-maximal concentration of Exendin-4 (0.1 nM) was co-administered with increasing concentrations of Rg1, Rg2, or Rg3 (Figure 2d). Luciferase-mediated light emission progressively decreased in a concentration-dependent manner. While all three compounds demonstrated an inhibitory trend at elevated concentrations, Rg3 exhibited the most pronounced suppression of receptor signaling, followed by Rg2, whereas Rg1 had a negligible effect.

This specific pattern, characterized by an absence of non-specific cellular activation in the null-background control (Figure 2b), an absence of direct agonism (Figure 2c), and selective, concentration-dependent functional inhibition in the presence of agonist stimulation (Figure 2d), is consistent with the computational data described below. Together with the MM-GBSA binding-energy calculations, these functional data support the hypothesis that dietary ginsenosides, particularly Rg3, negatively modulate human GLP-1R signaling, potentially through an allosteric.

### 2.2. Binding Site Identification and Molecular Docking Results

**Figure 3:**
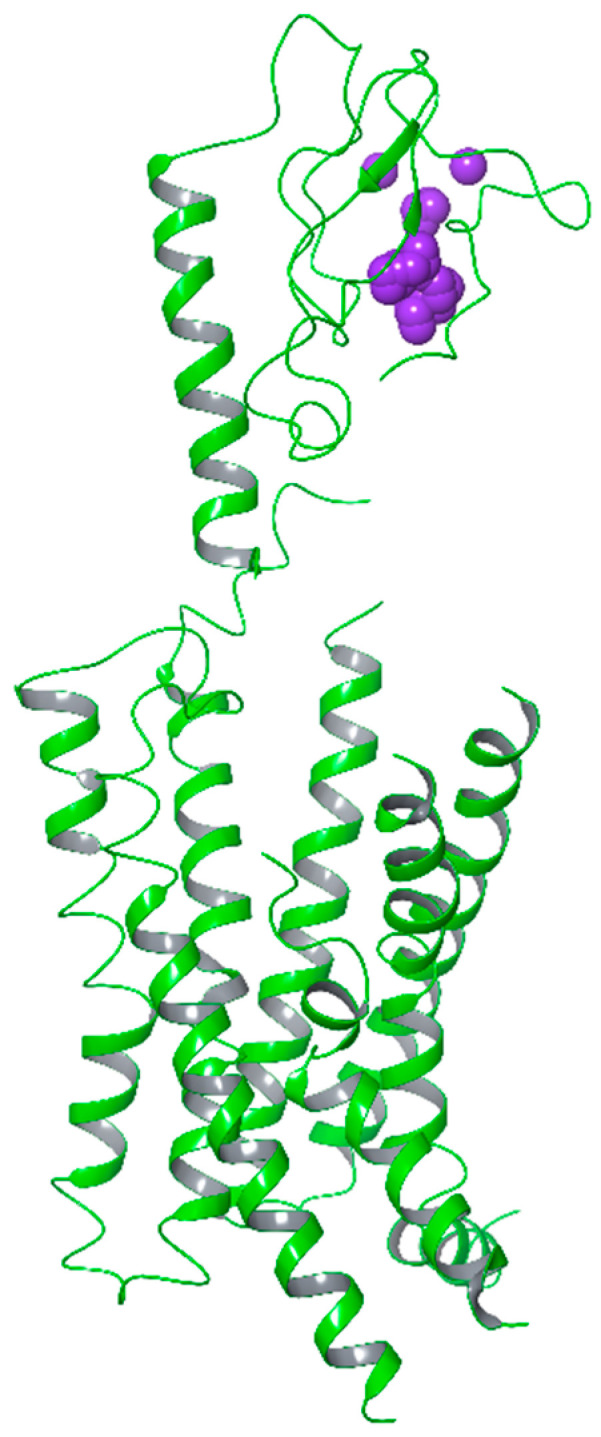
SiteMap-predicted a previously undiscussed extracellular binding pocket (ECP) on GLP-1R. The receptor is shown in green (ribbon representation), and the predicted binding site is highlighted in purple (space-filling representation). This figure was drawn using Schrodinger’s Maestro software (Version Schrodinger2022-2) (https://www.schrodinger.com/platform/products/maestro/ (accessed on 21 June 2025)).

Recognizing the distinct size and physicochemical properties of phytochemicals in comparison with GLP-like peptides, we searched GLP-1R for potential binding sites favorable for phytochemical binding beyond its orthosteric site. This broadened approach is supported by recent computational studies that have explored phytochemical interactions across multiple receptor regions, including non-canonical binding sites, to uncover novel modes of bioactive engagement [23,24,25]. To this end, we searched the 7LLL GLP-1R crystal structure for potential binding site using Schrodinger’s SiteMap tool (Version Schrodinger2022-2) [26], and identified a previously undiscussed binding pocket on the protein surface located at the N-terminal extracellular domain (Figure 3).

### 2.3. Molecular Docking and MM-GBSA Calculations

To assess the binding capabilities of the three ginsenosides considered in this work, molecular docking was conducted for Rg1, Rg2, and Rg3 against the ECP. Subsequently, binding free energy calculations were performed using the MM-GBSA method to evaluate the thermodynamic stability of each ligand–receptor complex.

All three compounds exhibited favorable binding scores to the potential binding site. Comparison of the MM-GBSA binding free energy values for the three molecules at ECP reveals notable differences in ligand binding stability (Table 1), suggesting differential binding preferences at this location. Rg3 demonstrated a strong binding energy (−46.31 kcal/mol), RG2 showed a moderately reduced ΔG (−37.01 kcal/mol) and Rg1 exhibited a significantly weaker binding (−20.08 kcal/mol). This pronounced predicted disparity at ECP which is in accord with the experimental findings, may indicate a selective affinity of Rg3 and to a lesser extent Rg2 for the N-terminal region, potentially implicating it as an allosteric site for ginsenosides binding, with ligand-specific interaction patterns.

**Figure 4:**
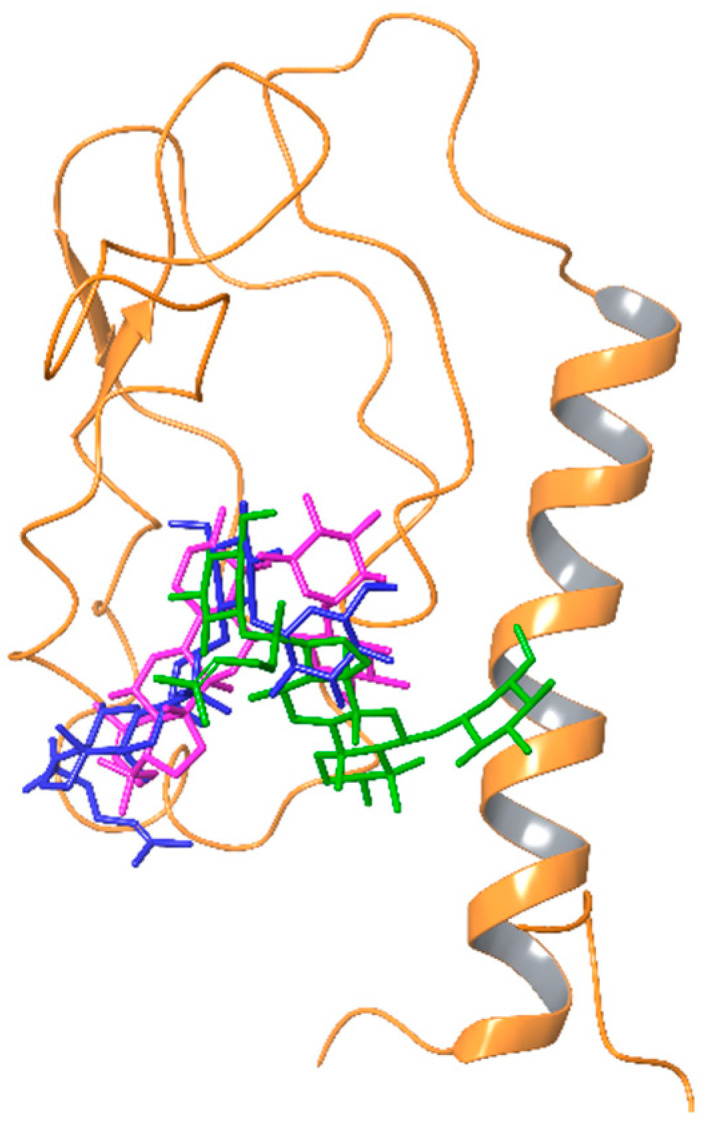
Binding poses of Rg1 (green), Rg2 (pink), and Rg3 (blue) at ECP of GLP-1R (orange). This figure generated using Schrodinger’s Maestro software (https://www.schrodinger.com/platform/products/maestro/ (accessed on 21 June 2025)).

Figure 4 depicts the three-dimensional binding orientation of Rg1, Rg2, and Rg3 within the predicted binding site of GLP-1R.

Within the ECP, which is located at the N-terminal region, Rg2 (pink) and Rg3 (blue) adopt a similar, horizontally aligned pose along the surface groove, indicating a possible common interaction pattern. However, Rg1 (green) is positioned differently, and engages with a separate region of the receptor.

## 3. Discussion

The emerging view of the gut as a sensory organ naturally leads to the question of how localized signals within the intestinal epithelium influence downstream metabolic regulation. The gut epithelium contains specialized enteroendocrine cells that sense nutrients and microbial metabolites, generating cellular responses to chemical stimuli. While traditional views focus on slow hormonal pathways or faster local neural routes linking the intestinal interface to metabolic centers [11,12], the precise direct receptor mechanisms remain an active area of investigation.

It is well established that many dietary phytochemicals, such as saponins and polyphenols, are poorly absorbed in the upper gastrointestinal tract. As a result, these compounds are delivered to the colon largely unmetabolized, resulting in relatively high concentrations both locally and during discrete time windows following meals and transit time in the intestine [27,28]. This pharmacokinetic profile distinguishes them from most endogenous signaling molecules and suggests that the colon may be a primary site for their bioactivities. Given the crucial role that colon cells expressing GLP-1Rs play as key local mediators of metabolic signaling, the GLP-1R represents an important therapeutic target, particularly regarding its modifiable ligand-binding capacities. Recent research shows that dietary phytochemicals and small molecules can allosterically modulate GLP-1R binding and signaling, supporting the exploration of GLP-1R as a target for novel plant-derived therapeutics [29,30,31]. Homology modeling and conformational sampling of the transmembrane domain of human GLP-1R have been used to predict an allosteric binding site and guide in silico screening for nonpeptidic ligands, yielding small-molecule agonists with activity in cAMP reporter assays and insulin secretion and demonstrating that GLP-1R can be regulated allosterically by druglike small molecules [4,5].

To assess whether ginsenosides modify ligand binding to GLP-1R, we used the functional luciferase assays and found that Rg3 and its analogs indeed decreased GLP-1R activity in a dose-dependent manner when co-administered with the agonist Exendin-4. This pattern suggests that these compounds that act as agonists or PAMs at taste receptors may instead function as functional antagonists or negative allosteric modulators (NAMs) by blunting receptor activation induced by a canonical ligand. While most studies have focused on enhancing GLP-1R signaling, the identification of natural NAMs may offer new avenues for modulating GLP-1R under conditions where attenuation is desirable, for example, to mitigate overstimulation or receptor desensitization.

Our in silico analysis has identified a previously undiscussed binding site on the GLP-1R surface. This region corresponds to a putative extracellular (N-terminal) allosteric site. Importantly, an analysis of multiple Class B1 GPCR structures indicates that the spatial characteristics of this N-terminal extracellular pocket are structurally conserved across nine other receptors within this class covering multiple families including Calcitonin, Corticotropin-releasing factor, Glucagon, and VIP and PACAP (PDB codes, 6UAV, 7TRY, 8F2A, 7DTY, 6LML, 6M1I, 6WI9, 7VQX). This baseline structural conservation suggests that the pocket represents an inherent architectural feature rather than an isolated configuration unique to the GLP-1R and hints that similar extracellular cavities may be present in related metabolic and endocrine receptors, serving as potential sites for localized chemical interactions. Systematically mapping this shared consensus pocket across the wider Class B family remains a subject for separate future structural and computational modeling investigations.

Ginsenoside Rg3 exhibited high affinity to the identified ECP, with particularly favorable MM-GBSA energies (−46.31 kcal/mol). These findings are consistent with previous reports that described the flexibility of class B GPCRs to accommodate ligands in both orthosteric and allosteric positions [5,6]. Encouragingly, the computational results (Glide XP Gscore and MM-GBSA energies) across this site mirror the experimental findings, both suggesting Rg3 as the strongest GLP-1R binder followed by Rg2 and Rg1 (Figure 2 and Table 1).

The structural selectivity observed among the three ginsenosides also warrants discussion. Rg1 showed significantly weaker binding at ECP, both energetically and as reflected in its binding mode, adopting a distinct binding orientation compared to Rg2 and Rg3. This suggests that even subtle variations in sugar moiety positioning on the triterpenoid core may substantially influence binding dynamics, a phenomenon previously described for other ginsenosides interacting with membrane proteins [20,22].

In the context of the present study, we acknowledge that docking scores and related computational estimates, such as MM-GBSA, cannot replace experimentally determined activity data or structure–activity relationship analyses. Although these methods provide thermodynamic approximations that can assist in early-stage compound prioritization, their results must be interpreted with caution due to inherent limitations. Similarly, while HEK293 cells provided a controlled system for investigating receptor activation, they lack the physiological context of gut tissues that express GLP-1R.

Our findings with ginsenosides and the GLP-1R prompt broader considerations of how diverse phytochemicals might interact with previously unrecognized extracellular or extramembrane domains on intestinal, gut, and colonic receptors. Such hypothesized interactions are supported by two key aspects of phytochemical behavior in the human gut. As noted above, many dietary phytochemicals are poorly absorbed and therefore reach the colon at high concentrations [27,28]. This pharmacokinetic profile distinguishes them from most endogenous signaling molecules and suggests that the colon may be a primary site for their bioactivity. Second, the physicochemical properties, hydrophilicity, glycosylation, low molecular weight, and rigid polycyclic structures of phytochemicals differ markedly from typical GLP-1R ligands. These features may limit phytochemicals’ ability to penetrate cell membranes or interact with transmembrane or cellular domains, while making them more accessible to extracellular regions of receptors. Recent computational and molecular dynamics studies support this, showing that such phytochemicals preferentially dock with extracellular domains rather than canonical orthosteric or intramembrane sites [32,33].

Findings from advanced structural biology and high-throughput screening efforts further reinforce this perspective [32,33]. Many cell surface receptors, including GPCRs and receptor kinases, possess complex extracellular domains that can serve as platforms for diverse ligand interactions, including structurally divergent dietary compounds [13]. These often poorly characterized domains could provide binding sites for phytochemicals and alter receptor functions in ways not yet fully understood. Thus, the interaction of dietary phytochemicals with previously unrecognized extracellular receptor domains may represent a physiologically relevant mechanism for modulating gut signaling.

Such interactions enable the local gastrointestinal environment to selectively sense and respond to elevated concentrations of structurally varied dietary phytochemicals, allowing potential biological benefits to be harnessed while minimizing risks of cellular overstimulation or toxicity. By leveraging advanced methodologies for mapping extracellular protein–ligand interfaces, it becomes clear that we can systematically explore these potential, putative direct interactions with intestinal receptors to fully explain their molecular modes of action. As the broader significance of these localized pathways continues to gain scientific recognition, characterizing these discrete receptor-level interfaces establishes a definitive framework for deciphering the precise mechanisms through which targeted dietary factors regulate gut signaling and metabolic health.

## 4. Methods

### 4.1. Luciferase Assay

HEK293 and COS7 cells (ATCC, Rockville, MD, USA) were used for the luciferase assay. The human GLP-1R (GenBank no. NP_002053) was cloned into the pcDNA3.1expression vector (Zeo-; Invitrogen, San Diego, CA, USA) under the CMV promoter. The plasmid was transformed into *E. coli* DH5α, grown overnight in LB medium with 100 µg/mL ampicillin, and extracted using a midi-prep kit (Invitrogen by Thermo Fisher Scientific, Vilnus, Lithuania).

Cells were maintained in DMEM with 10% FBS, 1% glutamine, 100 U/mL penicillin, and 100 µg/mL streptomycin (IMBH, Bet HaEmek, Israel) at 37 °C in 5% CO_2_ till confluent. Twenty-four hours prior transfection, cells were seeded at a 1:6 ratio using Trypsin (IMBH, Bet HaEmek, Israel). Co-transfection was performed using jetOPTIMUS (Polyplus, Graffenstaden, France) with 5 µg of GLP-1R plasmid and 5 µg of pCRE-LUC reporter plasmid (PROMEGA, Madison, WI USA) per plate. After overnight incubation, cells were replated in 96-well plates and starved in DMEM with 0.5% BSA for 24 h.

Cells were then treated with Exendin-4 (APExBIO, Houston, TX, USA) or test ligands (0.01 pM–0.01 µM), with or without 100 nM Exendin-4. After 6 h, 35 µL lysis buffer (Lysis 5× reagent, IMBH, Bet HaEmek, Israel) was added, and samples were frozen at −20 °C overnight. Luminescence was measured using a luciferin-ATP mix (1 M tris acetate, 1 M Mg acetate, and luciferin [Sigma Aldrich, St. Louis, MO, USA]; Promax, Promega, Madison, WI, USA) and a luminometer (Promax, Promega, Madison, WI, USA). Data were analyzed with GraphPad Prism 10.

Luciferase activity values represent the ratio between the luminescence signal measured for each treatment and the baseline signal obtained with Exendin-4 at 0.1 nM. Ginsenosides were evaluated across multiple concentrations. Lower ratio values indicate a greater inhibitory effect of the tested compound on GLP-1R–mediated signaling in HEK293 cells.

### 4.2. Molecular Modeling of Rg1, Rg2, and Rg3

The crystal structure of human GLP-1R (PDB ID: 7LLL), in complex with the peptide agonist Exendin-4, was used for docking of the three ginsenosides considered in this work. This structure corresponds to the active state of GLP-1R and was selected due to its relevance to the experimental studies performed in this work which tested the effect of the ginsenosides on the Exendin-4 mediated activation of GLP-1R. The structure contains five chains, from which only chain R, representing GLP-1R without associated G-proteins, was retained to reduce system complexity. First, the structure was processed by Schrodinger’s Protein preparation Wizard [34] to add hydrogen atoms, complete missing residues and/or sidechains and set correct protonated states to titratable residues at pH = 7. Next the processed structures was subjected to Schrodinger’s SiteMap [26] to identify druggable binding sites.

Prior to docking, the three ginsenosides were prepared by LigPrep as implemented in Maestro ((Version Schrodinger2022-2), New York, NY, USA). The receptor grid for GLP-1R was generated by specifying the binding site pockets, which were identified by SiteMap. Once the receptor grids were generated, the three ligands were docked to the receptor using Glide “Extra precision mode” (XP) docking protocol [35]. The docked conformers were evaluated using Glide (G) Score. Finally, the lowest energy poses for each ligand in each binding site were re-scored by Schrodinger’s MM-GBSA procedure [36] to obtain more accurate binding free energy estimates.

## 5. Conclusions

In conclusion, this study integrates computational modeling with functional cell-based assays to propose a novel mode of action for unabsorbed dietary phytonutrients at the human GLP-1R. Using differential luciferase activity as a sensitive marker for receptor inhibition, we demonstrated that the three structurally related ginsenosides exert distinct effects on GLP-1R signaling, consistent with their differential predicted binding at the newly identified N-terminal domain site. These results suggest that phytochemicals can exert significant physiological activity through direct interaction with the receptor’s extracellular domain, a mechanism of particular importance in the distal gut, where these compounds reach high local concentrations. Overall, this work suggests the extracellular domains of GLP-1R and perhaps of other intestinal receptors as potential mediators of dietary signaling and as critical targets for future research aimed at validating this non-systemic mode of action. Such investigations offer a strategic venue for the rational discovery of natural allosteric modulators among the wide array of unabsorbed phytonutrients known to influence metabolic health.

## Figures and Tables

**Table 1:** MM-GBSA Binding Free Energy, XP GlideScore Values and Luciferase Activity for Rg1, Rg2, and Rg3 at Predicted GLP-1R Binding ECP.

Rgs	MM-GBSA ΔG (kcal/mol)	XP Gscore (kcal/mol)	Luciferase Activity * (Ratio)
3	−46.31	−6.84	0.24
2	−37.01	−6.58	0.47
1	−20.08	−6.04	0.71

* Ginsenosides were evaluated across multiple concentrations, with 0.1 mM exhibiting the most pronounced activity, therefore these values are reported in the table.

## Data Availability

The original contributions presented in this study are included in the article/Supplementary Materials. Further inquiries can be directed to the corresponding author.

## Supplementary Materials

The following supporting information can be downloaded at: https://www.mdpi.com/article/10.3390/ijms27125630/s1.
