# Supplementary material for: Differential Modulation of GLP-1R by Dietary Ginsenosides Points to a Putative Extracellular Allosteric Site
Source: Int J Mol Sci. 2026 Jun 22;27(12):5630. doi: 10.3390/ijms27125630 (PMC13299201; doi:10.3390/ijms27125630)
Supplement: Supplementary file 1 [file ijms-27-05630-s001.zip › ijms-4317218-supplementary.pdf]

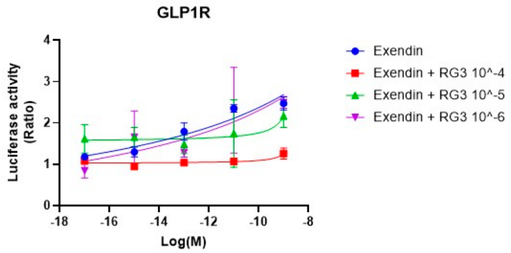

Supplementary Figure S1. Preliminary COS7 optimization of GLP-1R-mediated CRE-luciferase signaling. COS7 cells transiently expressing human GLP-1R and the CRE-luciferase reporter were treated with increasing concentrations of Exendin-4 alone or with Exendin-4 co-administered with Rg3 at the indicated concentrations. The data show the preliminary assay optimization performed in COS7 cells before selecting HEK293 cells for the definitive profiling shown in Figure 2.
